# Cronkhite-Canada Syndrome Associated With Superficial Esophageal Carcinoma: A Case Report and Literature Review

**DOI:** 10.3389/fmed.2022.855336

**Published:** 2022-03-25

**Authors:** Yan Zhao, Fujing Lv, Xun Yang, Yongjun Wang, Shutian Zhang, Peng Li

**Affiliations:** Department of Gastroenterology, Beijing Friendship Hospital, Capital Medical University, National Clinical Research Center for Digestive Diseases, Beijing Digestive Disease Center, Beijing, China

**Keywords:** Cronkhite-Canada syndrome, esophageal carcinoma, case report, endoscopic surveillance, gastrointestinal malignancy

## Abstract

**Introduction:**

Cronkhite-Canada syndrome is a rare disease characterized by generalized gastrointestinal polyposis, alopecia, skin pigmentation, and onychotrophia with no generally recognized mechanism of pathogenesis. There is a tendency of malignant transformation or coexistence of gastrointestinal malignancies in patients with Cronkhite-Canada syndrome.

**Case Description:**

The patient was a 67-year-old man who complained of dyspepsia, hair loss, skin hyperpigmentation, and pedal edema. Lab tests showed hypoalbuminemia. Endoscopic findings included superficial esophageal carcinoma and numerous polyps in the stomach, duodenum, and colon. The patient was treated with endoscopic submucosal dissection for the esophagus lesion, endoscopic mucosal resection for colon polyps, and glucocorticoids for Cronkhite-Canada syndrome.

**Conclusion:**

Esophagus cancer is a rare comorbidity of Cronkhite-Canada syndrome. Endoscopic examination and surveillance are critical for patients with Cronkhite-Canada syndrome for malignant gastrointestinal tumors.

## Introduction

Cronkhite-Canada syndrome is a rare disease characterized by generalized gastrointestinal polyposis, alopecia, skin pigmentation, and onychotrophia first described by Leonard W. Cronkhite Jr. and Wilma Jeanne Canada in 1955 (1). A nationwide survey conducted by Oba et al. reported 473 patients with Cronkhite-Canada syndrome throughout Japan in 2017. The prevalence rate was 3.7 per 100,000 and the male-to-female ratio was 1.1:1. Sessile and pedunculated polyps spread widely in the stomach, duodenum, small intestine, and colon. The esophagus, however, is rarely involved in Cronkhite-Canada syndrome (2, 3). Additionally, diffuse thickening of the gastrointestinal (GI) tract mucosa with hypertrophic rugae is often noticed, particularly in the upper GI tract (3, 4).

The contemporary occurrence of gastrointestinal carcinoma in patients with Cronkhite-Canada syndrome is rare (5). There have been reports of malignant comorbidities including gastric carcinoma, esophageal carcinoma, colon carcinoma, and cholangiocarcinoma (5–11). Unfortunately, however, the mechanism of malignant transformation in Cronkhite-Canada syndrome is not yet known clearly, but the presence of autoimmune-related IgG4 antibody in polyps and response to immunosuppression therapy suggest that Cronkhite-Canada syndrome might be an autoimmune-related disease (12). Abnormal gene expression, such as inhibin beta A, might also contribute to the pathogenesis of Cronkhite-Canada syndrome (13).

Here we present a case of a patient with Cronkhite-Canada syndrome and contemporary early esophageal carcinoma as well as a precise review of relevant works of literature, in order to raise the attention on malignant gastrointestinal comorbidities.

## Case Description

### Chief Complaint

A 67-year-old man who complained of dyspepsia, hair loss, skin hyperpigmentation, and pedal edema was referred to Beijing Friendship Hospital by his preliminary care physician.

### History of Present Illness and Physical Examination

**Figure 1:**
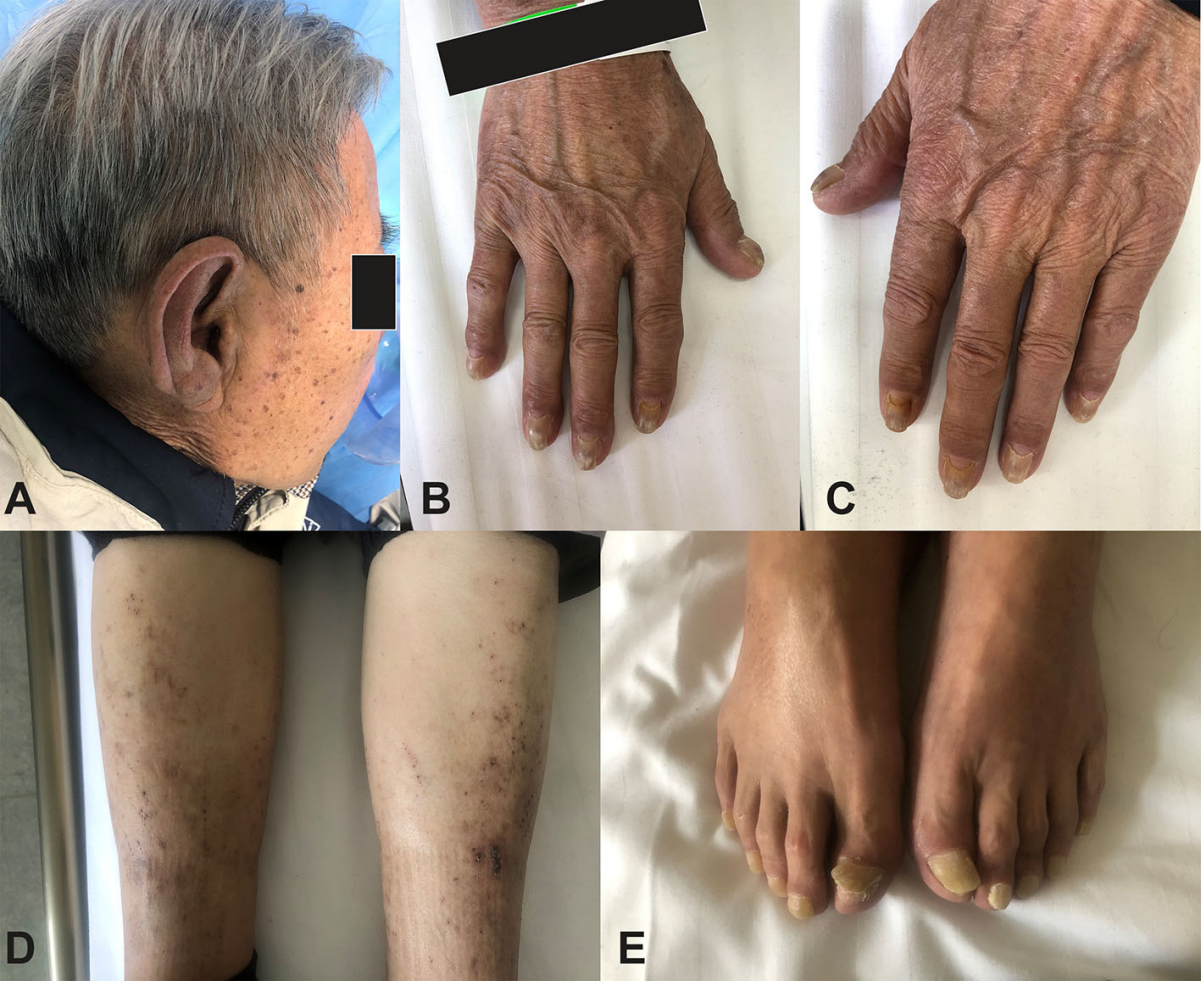
Clinical manifestation of the patient. **(A)** Skin hyperpigmentation and alopecia. **(B,C)** Skin hyperpigmentation and onychodystrophy. **(D)** Hyperpigmentation and lower limb edema. **(E)** Onychodystrophy (toenail).

The patient complained that the symptoms started several months ago but were ignored by the patient and not evaluated by physicians. However, the symptoms deteriorated in the past few months, which brought him to his primary care physician who referred him to our department. The patient reported no dysphagia or odynophagia. Physical examination revealed alopecia, onychotrophia, and pitting edema in lower extremities (Figure 1).

### Past Medical History and Family History

Past medical history included hypertension, which was diagnosed 3 years before admission and controlled well by amlodipine 2.5 mg per day.

The patient eats a balanced diet, hardly any intake of hot food, and occasional smoking and drinking. His place of birth and living was not an endemic region of esophagus squamous cell carcinoma. Both of his parents died of natural causes and his two sisters had not been diagnosed with cancer.

### Laboratory and Imaging Examinations

**Figure 2:**
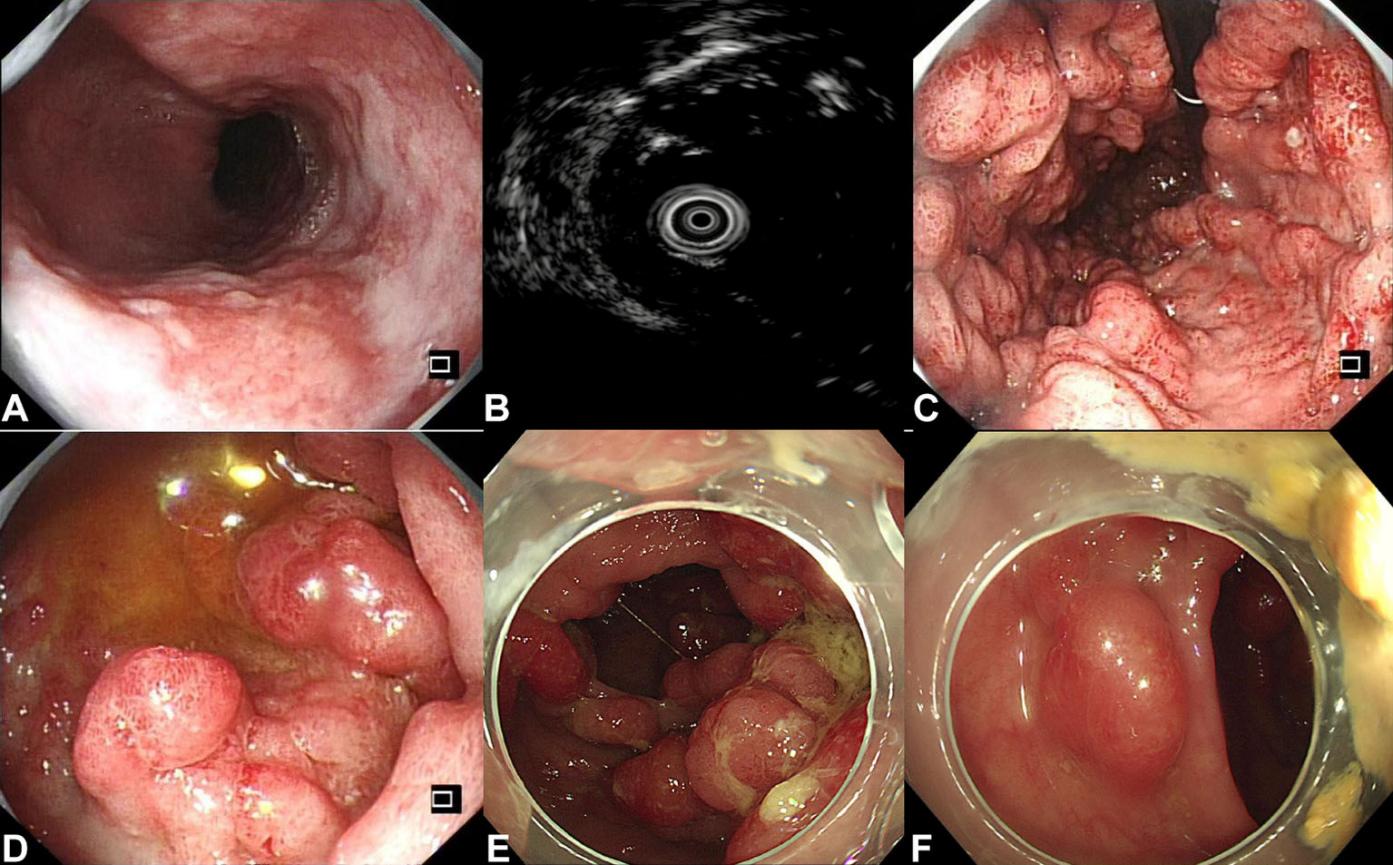
Endoscopic images. **(A)** Esophageal lesion (at the 6 o'clock direction, size 2.2 × 1.0 cm, type Paris 0-IIb). **(B)** Endoscopic ultrasound (EUS) confirmed the lesion originated from the mucosa layer. **(C)** Engorgement and hyperemia of gastric folds, as well as multiple polyps in the stomach **(C)**, duodenum **(D)**, and colon **(E,F)**, were noticed.

Serum albumin level was 28.7 g/L (reference range: 40–55 g/L). Esophagogastroduodenoscopy (EGD) revealed a Paris 0-IIb mucosal lesion occupying 1/3 esophageal circumference originating from the mucosa layer, which was confirmed by magnifying endoscopy and endoscopic ultrasound. During a colonoscopy, numerous polyps were discovered spreading from the distal ileum to the rectum (Figure 2).

### Endoscopic Treatment and Histological Findings

**Figure 3:**
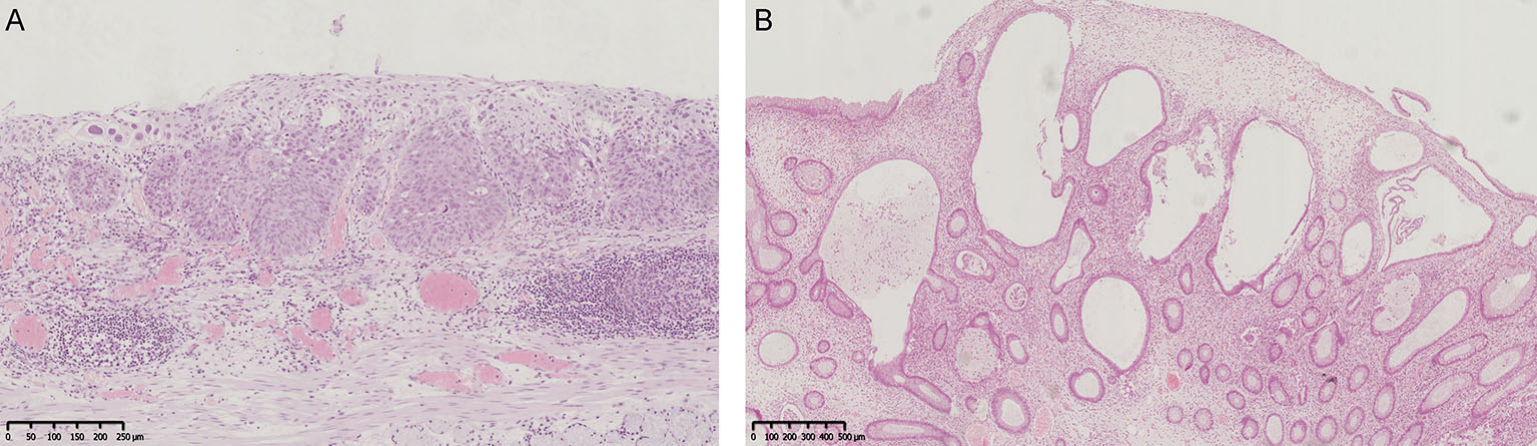
Histological images. **(A)** Esophageal lesion. Squamous cell carcinoma. Atypia cells with abundant eosinophilic cytoplasm and keratinization were observed. Mt, type Paris 0-IIb, 22 × 10 mm, SCC, pT1a-LPM, INFa, ly0, v0, pHM0, pVM0, pR0, pCurA (Middle third of esophagus, flat type, size: 22 × 10 mm, squamous cell carcinoma, tumor invades lamina propria mucosae, infiltration pattern A, no lymphatic invasion, no venous invasion, clear horizontal margin, clear vertical margin, no residual tumor, complete removal of the tumor). **(B)** Colon juvenile polyp. Prominent, cystically dilated glands and inflammatory stroma were observed.

The patient was treated with endoscopic submucosal dissection (ESD) for the esophageal lesion and endoscopic mucosal resection (EMR) for colon polyps. Histological examination revealed that the lesion of the esophagus was squamous cell carcinoma. Hyperplastic polyps were identified in the gastric specimens. Polyps in the colon contained juvenile type polyp and inflammatory polyps (Figure 3).

### Final Diagnosis and Pharmacological Treatment

Based on the presentation of alopecia, skin hyperpigmentation, onychotrophia, and gastrointestinal polyposis including juvenile type polyps, the diagnosis of Cronkhite-Canada syndrome was established. Prednisone 40 mg (0.67 mg per kilogram of body weight) per day was also administered, as well as nutrition supplementation and probiotics.

### Outcome and Follow-Up

The patient was discharged with a better appetite, alleviated edema, and no sign of gastrointestinal hemorrhage. The patient was followed for a year and the symptoms were relieved with no recurrence of esophageal carcinoma. EGD and colonoscopy were conducted 6, 12, and 18 months after ESD, which revealed no sign of recurrence of esophageal squamous carcinoma, decreased density of gastrointestinal polyps, and healed GI mucosa.

## Discussion

Since first reported in 1955, Cronkhite-Canada syndrome has been recognized as a syndrome involving multiple systems (1), which was differentiated from other polyposis syndromes by distinct extraintestinal symptoms and widespread polyps throughout the GI tract, including stomach, small intestine, and colon (14). The existence of gastrointestinal cancer in patients with Cronkhite-Canada syndrome has been noticed recently. Yashiro et al. summarized known cases of Cronkhite-Canada syndrome reported between 1967 and 2002, concluding that among a total of 387 patients, 50 patients suffered from comorbidities of gastrointestinal malignancies, including 31 cases of colon cancer and 19 patients of gastric malignant neoplasia. Further analysis showed that male patients with Cronkhite-Canada syndrome were more susceptible to colorectal cancers (15). To detect any possible gastrointestinal malignancies in patients with Cronkhite-Canada syndrome, regular and meticulous endoscopic surveillance is necessary.

In our case, the patient suffered from both Cronkhite-Canada syndrome and esophageal carcinoma. Only a few cases with esophageal cancer were reported before (8, 16), which makes esophageal cancer associated with Cronkhite-Canada syndrome unusual. Ito et al. reported a 74-year-old Japanese man with Cronkhite-Canada syndrome associated with esophageal squamous cell carcinoma and gastric cancer, who was treated with chemoradiotherapy for the esophageal lesion, surgical resection for gastric cancer, and glucocorticoids for Cronkhite-Canada syndrome (8). Esophageal papilloma is another abnormality originating from squamous cells in patients with Cronkhite-Canada syndrome (17, 18). Considering that esophageal carcinoma and papilloma both originate from squamous cells of the epithelium, esophageal squamous cells might be a susceptible target in Cronkhite-Canada syndrome.

It must be noticed that the esophagus was spared from inflammation in this patient, which was similar to the case reported by Ito et al. (8). It is well-known that inflammation mucosa is predisposed to neoplastic changes, but whether this theory correctly applies to the condition of Cronkhite-Canada syndrome is worth discussion. The mechanism of pathogenesis and GI malignant transformation in patients with Cronkhite-Canada syndrome should be further explored.

As for the treatment of Cronkhite-Canada syndrome, there have been no universally accepted guidelines and the regimens are highly variable depending on the clinical characteristics of each individual (19). Generally, immunomodulant therapy is recommended and adopted as a first-line choice, including glucocorticoids, immunosuppressants, and monoclonal antibodies targeting key cytokines in the inflammatory response (20–23). In our study, the patient received glucocorticoids as initial treatment and responded well. Specifically, the density of polyps decreased and mucosa between polyps appeared to be less edematous and congestive. Additionally, symptoms were relieved and albumin levels increased. The efficacy of steroids, to some degree, proved that autoimmune dysregulation might have contributed to the pathogenesis of Cronkhite-Canada syndrome. The dosage and course of steroid treatment must be carefully managed and adverse effects should be frequently monitored.

The treatment regimen also contained endoscopic resection of gastrointestinal polyps. It must be noted that due to the high density and wide distribution of polyps, it is impossible to resect all polyps during one procedure. Biopsies and resections of potentially malignant polyps (chromoendoscopy and magnifying endoscopy might be applied) should be prioritized. Apparently, more biopsies and resections mean a more accurate and comprehensive understanding of the histology of polyposis in Cronkhite-Canada syndrome, which, however, might cause more damage to the gastrointestinal tract. Therefore, a delicate balance must be established to ensure maximum detection of potentially malignant lesions with the least harm.

## Conclusion

Cronkhite-Canada syndrome is gastrointestinal polyposis with extraintestinal manifestations and the potential tendency of malignant comorbidities or transformation. Endoscopic examinations, biopsy, and polyp resection play a crucial role in the diagnosis, treatment, and surveillance of Cronkhite-Canada syndrome, with special focus on any suspected malignancy. Regimens including corticosteroids and endoscopic treatment might be applied with individualized treatment protocols.

## Data Availability Statement

The original contributions presented in the study are included in the article/supplementary material, further inquiries can be directed to the corresponding authors.

## Ethics Statement

The studies involving human participants were reviewed and approved by Ethic Committee of Capital Medical University Beijing Friendship Hospital. The patients/participants provided their written informed consent to participate in this study.

## Author Contributions

YZ, FL, and XY were responsible for the treatment of the patient during inpatient care. SZ and PL performed the endoscopic treatment and surveillance and conceptualized the case report. YW was responsible for following up the patient. All authors contributed to writing and revising the manuscript.

## Conflict of Interest

The authors declare that the research was conducted in the absence of any commercial or financial relationships that could be construed as a potential conflict of interest.

## Publisher's Note

All claims expressed in this article are solely those of the authors and do not necessarily represent those of their affiliated organizations, or those of the publisher, the editors and the reviewers. Any product that may be evaluated in this article, or claim that may be made by its manufacturer, is not guaranteed or endorsed by the publisher.
